# The physical impact of migraines on female chiropractic patients: A qualitative study

**DOI:** 10.4102/hsag.v28i0.2283

**Published:** 2023-10-25

**Authors:** Ashalya Pirthiraj, Raisuyah Bhagwan

**Affiliations:** 1 Department of Chiropractic, Faculty of Health Sciences, Durban University of Technology, Durban, South Africa; 2 Department of Community Health Studies, Faculty of Health Sciences, Durban University of Technology, Durban, South Africa

**Keywords:** migraines, female migraineurs, physical impact, physical functioning, triggering factors, contributing factors, chiropractic

## Abstract

**Background:**

Migraines are highly prevalent among the female population and have a significant burden on one’s quality of life and physical functioning.

**Aim:**

The study explored the physical impact and contributory factors of migraines on women and their experience of chiropractic treatment for migraine pain management.

**Setting:**

The study was conducted in the eThekwini region of KwaZulu-Natal, South Africa.

**Methods:**

The study used a qualitative descriptive design and adopted purposive sampling. The data were collected through 12 semi-structured interviews, between March and September 2021, and analysed using thematic analysis.

**Results:**

The first theme that emerged focused on the physical effects of migraines. The second theme related to the factors that contributed to migraines. The third theme that emerged focused on chiropractic treatment for migraines.

**Conclusion:**

The majority of the participants experienced chronic migraines and migraines without aura. The participants felt incapacitated and experienced debilitating physical effects with their migraines. The study highlighted that chiropractic treatment was favourable among the female population in improving the quality of life and reducing the severity, disability, duration of suffering and frequency of migraines. It was revealed that chiropractic was the preferred non-pharmacological approach for migraine treatment, as it proved to be a beneficial and effective treatment for migraine pain management.

**Contribution:**

The findings contribute to a greater awareness of chiropractic as an effective evidence-based treatment approach for migraine pain management, which may be beneficial to migraineurs and healthcare practitioners.

## Introduction

A migraine headache is a primary headache disorder that is characterised by reversible systemic or neurological symptoms (Dodick 2018:1315). Migraine headaches are one of the world’s most debilitating headaches, ranking seventh among the 10 leading causes of years lived with disability worldwide (Vos et al. 2016:1545). Migraines affect nearly 56 million people in Africa and are prevalent among people of all age groups (Woldeamanuel, Andreou & Cowan 2014:1). However, it is most prevalent globally among young and middle-aged women (Stovner et al. 2018:954). Migraines are three times more common in females than males, with the peak prevalence between 25 and 55 years of age (Dodick 2018:1317; Connor et al. 2021:1). Migraines were the most common headache type among South African university students, with the majority of migraineurs being females (Basdav, Haffejee & Puckree 2016:1680; Prangley 2010:49).

Migraine without aura presents with photophobia, phonophobia, nausea or vomiting (International Headache Society 2018:19). Migraines last 4–72 h (when untreated), with an average of 24 h duration per attack (Dodick 2018:1315). Migraine with aura presents with ‘unilateral fully reversible visual, sensory or other central nervous system symptoms that usually develop gradually [aura] and are usually followed by headache and associated migraine symptoms’ (International Headache Society 2018:20). The aura may last between 5 and 60 min.

Although migraines are highly prevalent and have a significant burden on the quality of life, they are underdiagnosed and undertreated (Befus et al. 2018:1). Because of the comorbidity of musculoskeletal complaints and migraines, chiropractic is a potentially promising evidence-based non-pharmacological approach to migraine management (Connor et al. 2021:2; Rist 2019:533; Wayne et al. 2020:1). Chiropractic is:

[*A*] health profession concerned with the diagnosis, treatment and prevention of mechanical disorders of the musculoskeletal system, and the effects of these disorders on the function of the nervous system and general health. (World Federation of Chiropractic 2001)

Chiropractic aims to reduce the overall burden of pain and optimises neuromusculoskeletal health by correcting postural strain and reducing muscle tension (Bernstein et al. 2019:2). Chiropractic treatment was documented to reduce the migraine severity, frequency, duration of suffering and associated disability among migraineurs (Bernstein et al. 2019:4; Bryans et al. 2011:274; Clar et al. 2014:2; Rist 2019:532). An international study conducted among women with migraine who received chiropractic care found that following chiropractic treatment, the ‘participants consistently stated that the treatment had increased their appreciation for the complex interaction between stress, muscular tension, posture, and migraine’ (Connor et al. 2021:5). Furthermore, the aforementioned study contributed to greater awareness among patients towards the musculoskeletal contributory factors as triggers of migraines. There are various contributing factors that can precipitate a migraine attack, and each individual’s triggers are different (Moore et al. 2017:519).

Most international studies on the impact of migraines were conducted quantitatively, with only a few studies using a qualitative approach (Banciu & Bouleanu 2018:27). Prior studies on migraines in South Africa have only used quantitative approaches to document the impact of migraines (Basdav et al. 2016; Du Preez 2004; Kleingeld 2016; Prangley 2010). A paucity of literature was revealed among patients’ experiences of migraine-related chiropractic treatment (Connor et al. 2021:2). Moore et al. (2020:2) suggested the need for more literature to understand the headache features within the migraine population and the role of chiropractic in their management. In the South African context, an empirical gap in the literature exists between the physical impact of migraines on women and their experience of chiropractic treatment for migraine pain management.

## Methods

### Study design

This study adopted a qualitative descriptive design that explored the physical impact and contributory factors of migraines on women and their experience of chiropractic treatment for migraine pain management.

### Setting

The study was conducted in eThekwini, KwaZulu-Natal, South Africa. Online video call interviews were primarily used for data collection to adhere to the COVID-19 protocols at the time of the study. Physical interviews were also conducted at a location that was convenient for the participants.

### Study population and sampling strategy

Women between 18 and 65 years of age who sought chiropractic treatment for migraine headaches in eThekwini, KwaZulu-Natal, were included in the study. Purposive sampling was used to recruit the participants.

Inclusion criteria:

- women between 18 and 65 years of age;
- women with episodic or chronic migraines, without aura or with aura;
- participants who received chiropractic treatment for their migraines; and
- those who consented to participate in the study.

Exclusion criteria:

- women between 18 and 65 years of age without migraines;
- participants who were diagnosed with other primary or secondary headaches;
- participants who did not receive chiropractic treatment for migraines; and
- participants who did not consent to participate.

### Data collection

Chiropractors in eThekwini were contacted telephonically to confirm their interest in the recruitment of their migraine patients as participants for the study. A request was made to trace and inform their patients, who met the inclusion criteria of the proposed study. Permission to recruit their migraine patients as participants for the study and to place a flyer in the waiting room of their practice to enable the recruitment of participants was obtained from each chiropractor. Those participants willing to participate contacted the researcher. No coercion was used to recruit participants in the study. The data were collected through 12 semi-structured interviews by the researcher (MTech Chiropractic) using an interview schedule until saturation was reached. Data saturation occurred with repetition of collected data and no new information was presented. Online semi-structured video call interviews and physical interviews were conducted between March and September 2021. Each interview lasted approximately 45 min. Descriptive field notes were taken during the interviews.

The interview schedule consisted of the following questions:

- Can you share with me when you began experiencing migraines?
- How often do you experience migraines and how many days per month?
- What are some of the factors that precipitate your migraines? What triggers your migraines? Probes: Diet, stress, hormonal changes.
- Can you share with me what your experience of a migraine is with regard to the physical symptoms and effects?
- What is your experience of chiropractic treatment for your migraines? How has chiropractic treatment helped you with managing your symptoms and pain?
- What do you like the most about chiropractic treatment in managing your symptoms and pain? What was most beneficial or helpful in your opinion?

### Data analysis

The interviews were audio recorded and subsequently transcribed after each interview. To ensure data accuracy, the transcripts and recordings were cross-checked multiple times. The data were analysed manually using thematic analysis to find emerging patterns, leading to the formation of main themes and sub-themes from the primary data.

### Trustworthiness

The model by Lincoln and Guba was used to guide trustworthiness and rigour for the study (Nowell et al. 2017:3). Credibility was ensured by member checking and theoretical triangulation, which assisted the research to reflect the opinions of the participants as accurately as possible. The study achieved confirmability by keeping an audit trail that detailed the process of data collection, data analysis and data interpretation, as well as the use of participants’ quotes to support interpretations. Transferability was upheld by providing sufficient information about the study for it to be generalised to other contexts. To ensure dependability, the research process was clearly documented, logical and traceable.

### Ethical considerations

The study received full approval from the Institutional Research Ethics Committee (IREC) in the Faculty of Health Sciences at the Durban University of Technology (IREC no. 173/20). Permission to recruit participants from chiropractic practices in eThekwini, KwaZulu-Natal was obtained. Consent for participation in the study was obtained by using a detailed letter of information and informed consent form, which were provided to each participant to sign before the interview. The participants were allowed to request further information before consenting and were made aware that their participation in the study was voluntary. Confidentiality was upheld as pseudonyms were used for the participants’ identities in the final write-up of the study. Furthermore, all data were stored appropriately (on a password-protected laptop and in a locked cabinet) during and after the study to maintain confidentiality.

## Results

Table 1 presents the demographic profile of the participants. Table 2 depicts the themes and sub-themes of the study. The first theme that emerged focused on the physical effects of migraines. The second theme related to the factors that contributed to migraines. The third theme that emerged focused on chiropractic treatment for migraines.

**TABLE 1:** Demographic profile of participants.

Participant	Age	Occupation
1	32	Sales representative
2	31	Internal sales
3	27	Business development officer
4	30	Quantity surveyor
5	38	Police official
6	25	Electrical engineer
7	44	Operations manager
8	51	Housewife
9	62	Housewife
10	45	Personal trainer
11	40	Hairdresser, beautician, teacher
12	29	Merchandising manager

**TABLE 2:** Themes and sub-themes.

Theme	Sub-themes
Theme 1: Physical effects	1.1 Symptoms of migraine 1.2 Impact on physical functioning and daily life
Theme 2: Factors contributing to migraines	2.1 Stress 2.2 Hormonal factors 2.3 Oral contraceptives 2.4 Dietary factors 2.5 Weather-related factors and lighting 2.6 Musculoskeletal factors 2.7 Sleep deprivation
Theme 3: Chiropractic treatment for migraines	3.1 Experiences of chiropractic treatment for migraine pain management 3.2 Perceptions of chiropractic treatment approaches for migraine management

### Theme 1: Physical effects

The first sub-theme described the participants’ migraine symptoms. Chronic migraines and migraines without aura were experienced by most participants. The pain was located in the frontal, suboccipital and temporal regions of the head with associated eye and neck pain. Most participants experienced unilateral pain, which was severe and excruciating. The pain character was described as heavy, throbbing and pounding. The following excerpts reveal the location, character and symptoms of migraine pain:

‘I get a throbbing sensation behind my eyes and behind my forehead. The pain runs from the front of my head all the way through to the back. Majority of the time it’s on one side.’ (Participant 2, Age 32, Sales representative)

‘I can only explain it as pressure.’ (Participant 3, Age 27, Business development officer)

‘The base of my skull, top of my head, the temples and a lot around my eye area.’ (Participant 4, Age 30, Quantity surveyor).

The symptoms experienced by the participants with migraine without aura were photophobia, phonophobia, hyperosmia, nausea, vomiting, loss of appetite, neck pain and light-headedness. The following excerpts reveal the associated symptoms experienced with migraine without aura:

‘[*T*]he sensitivity to light, sound and smell.’ (Participant 4, Age 30, Quantity surveyor)

‘I feel nauseous, very lightheaded.’ (Participant 8, Age 51, Housewife)

‘It’s vomiting, neck pain.’ (Participant 11, Age 40, Hairdresser)

The participants with migraine with aura experienced temporary changes in their vision, including photophobia, phonophobia, nausea, vomiting, and vertigo. The following excerpts reveal the symptoms of migraine with aura:

‘I experience issues with my depth perception. Light, noise and movement sensitivity are the three big warning triggers for me that a migraine’s going to hit. With the vestibular migraine, I usually have these dizzy episodes, with no nausea. I feel off-balanced. Then the pain will start. It will be noise sensitivity, light sensitivity, then nausea and then the throbbing pain. I couldn’t be in a room with anybody else because if they moved, it triggered the vertigo.’ (Participant 3, Age 27, Business development officer)

‘Hazy and black spots.’ (Participant 7, Age 44, Operations manager)

‘I used to see a zigzag and stars.’ (Participant 8, Age 51, Housewife)

The second sub-theme focused on the impact of migraines on physical functioning and daily life. The participants felt incapacitated and experienced debilitating effects during a migraine attack. They described that their life came to a standstill and were unable to complete daily activities:

‘I was incapacitated.’ (Participant 3, Age 27, Business development officer)

‘It’s probably the worst thing I’ve ever experienced.’ (Participant 12, Age 29, Merchandising manager)

‘I can’t concentrate.’ (Participant 9, Age 62, Housewife)

‘It’s exhausting.’ (Participant 1, Age 32, Sales representative)

‘I can barely manage the days that I have it.’ (Participant 8, Age 51, Housewife)

‘I felt it was quite debilitating because I couldn’t do anything.)

‘It’s very hard to sleep when you’re in that kind of pain.’ (Participant 10, Age 45, Personal trainer)

### Theme 2: Factors contributing to migraines

The sub-themes that emerged were hormonal factors, oral contraceptives, dietary factors, weather-related factors and lighting, musculoskeletal factors and sleep deprivation. All participants, except for one, described stress as a major contributing factor in triggering their migraines:

‘It depends how much stress I’m under. Stress is a major factor.’ (Participant 1, Age 32, Sales representative)

‘I have also noticed stress is a factor.’ (Participant 5, Age 38, Police official)

‘I think stress is a big one.’ (Participant 4, Age 30, Quantity surveyor)

Hormonal factors were found to trigger migraines, and most participants noticed a hormonal relationship between their migraines and menstruation. Nine of the 12 participants experienced migraines during their menstrual cycle. Six participants had migraine attacks either before or after menstruation, and two participants had attacks during menstruation. One participant had menstrual migraine with aura:

‘It happens at least three days before (my period starts). It stops just before the bleeding starts, then throughout the cycle there’s no migraines.’ (Participant 3, Age 27, Business development officer)

‘I used to get it a week before and after I had my period.’ (Participant 7, Age 44, Operations manager)

‘The aura was actually hormonal.’ (Participant 4, Age 30, Quantity surveyor)

One participant experienced severe migraines throughout her pregnancy and had a history of migraine with aura. The other two participants with children did not experience migraines during pregnancy. The excerpts below reflect the responses of migraine during pregnancy:

‘Throughout my pregnancy, every month I used to get it.’ (Participant 9, Age 62, Housewife)

‘I never had migraines when I was pregnant.’ (Participant 5, Age 38, Police official)

Migraines were triggered by oral contraceptives among three participants. Two participants experienced migraines after the hormone-free interval (placebo week), and one participant had migraine attacks every week on the combined oral contraceptive (COC), including the hormone-free interval:

‘It would happen after my placebo week. The auras were caused by the pill.’ (Participant 4, Age 30, Quantity surveyor)

‘It’s once I started with contraception that my migraines started. I was on the pill.’ (Participant 5, Age 38, Police official)

‘I didn’t know it was the pill that was also aggravating or causing the problems further.’ (Participant 7, Age 44, Operations manager)

The dietary contributory factors included inconsistent eating habits and dehydration. Participants stated that dairy products such as cheese, milk and chocolate triggered their migraines. Caffeine, pungent foods and red wine were also triggering factors. The excerpts below reflect the contribution of dietary factors, inconsistent eating habits and dehydration as triggering factors:

‘If I would have a glass of wine, if I didn’t eat it will (be triggered) and if I don’t have enough water.’ (Participant 1, Age 32, Sales representative)

‘The food that triggered it was cheese.’ (Participant 4, Age 30, Quantity surveyor)

‘If I eat spicy food, it triggers it.’ (Participant 5, Age 38, Police official)

‘Very salty foods.’ (Participant 2, Age 31, Internal sales)

‘Chocolate, dairy and caffeine.’ (Participant 12, Age 29, Merchandising manager)

Most participants reported that changes in weather, sunlight or bright lights are migraine-triggering factors. Fluorescent lights and humidity triggered migraines in one participant:

‘The sensitivity to light, like on overcast days I would not be able to go outside.’ (Participant 4, Age 30, Quantity surveyor)

‘Bright light, like the sun.’ (Participant 2, Age 31, Internal sales)

‘If I have to go out at night, bright lights from oncoming traffic.’ (Participant 9, Age 62, Housewife)

‘Fluorescent lights.’ (Participant 12, Age 29, Merchandising manager)

‘Cold wind, a change of weather.’ (Participant 9, Age 62, Housewife).

Participants were aware of the musculoskeletal factors that triggered their migraines. Poor posture, working long hours on the computer, bruxism or jaw tension, muscular pain, neck tension and physical work triggered their migraines:

‘Posture definitely was one of the big reasons for my migraine.’ (Participant 4, Age 30, Quantity surveyor)

‘I think the position I used to work in that affected it and caused it.’ (Participant 6, Age 25, Electrical engineer)

‘Possibly strain from working out; I suffer with TMJ (temporomandibular joint dysfunction). The jaw clenching triggers it.’ (Participant 1, Age 32, Sales representative)

‘Travelling used to trigger it a lot… muscular pain would trigger it.’ (Participant 4, Age 30, Quantity surveyor)

‘I think its overworking.’ (Participant 11, Age 40, Hairdresser)

A few participants reported sleep deprivation or inconsistent sleeping habits as migraine-triggering factors. Most participants slept earlier than usual or during their migraine attacks to cope with their pain.

‘What also could be contributing, with only getting a couple hours of sleep it’s also adding to the stress and the tension that I’m under.’ (Participant 7, 44, Operations manager)

‘I’ve noticed it does depend on whether I’m getting a lot of sleep or I’m not sleeping a lot.’ (Participant 12, 29, Merchandising manager)

### Theme 3: Chiropractic treatment for migraines

The first sub-theme related to the experiences of chiropractic treatment for migraine pain management. Participants revealed that chiropractic was their preferred approach to migraine management, as it proved to be a beneficial and effective treatment approach for their migraines compared to other treatments. Furthermore, chiropractic reduced the severity of their migraines, disability and the duration of suffering. Chiropractic reduced the frequency of migraines among some participants and improved their quality of life. The participants also stated that chiropractic released the migraine ‘pressure’ and relieved muscular tension:

‘When it comes to pain, it has reduced significantly after going to a chiropractor… the quality of life is so much better now. It’s one of the only things that helped me.’ (Participant 4, Age 30, Quantity surveyor)

‘If we can start treatment earlier, it reduced the need of medication, and the severity of the migraine. The recovery time is faster, and I don’t need to take as many pharmaceutical drugs than required. I would go to the chiropractor first, before taking medication, because it’s that much fast a relief.’ (Participant 3, Age 27, Business development officer)

‘I haven’t experienced as many severe headaches as I used to. It reduced drastically. I think it was how quickly I felt the relief of the pain.’ (Participant 6, Age 25, Electrical engineer)

‘I can take as much medicine as I want to and it just doesn’t help, and as soon as I go to the chiropractor it definitely helps.’ (Participant 12, Age 29, Merchandising manager)

The second sub-theme focused on the perceptions of chiropractic treatment approaches for migraine management. The participants stated that the most beneficial aspects of chiropractic for migraines were cervical spinal manipulative therapy (SMT), dry needling, soft tissue therapy, ischaemic compression, electro-modalities, cryotherapy, strapping and the advice on posture, strengthening exercises or stretches to manage the musculoskeletal complaints associated with their migraines. The excerpts below reveal the participants’ responses to the most beneficial aspects of chiropractic treatment for managing migraine pain:

‘My chiropractor has been amazing from the adjustments, from my pain management, also from the point of view that she listens. We chat about where the pain is, what’s caused the pain, how I’m feeling… I think that also helps, understanding what the patient is going through.’ (Participant 1, Age 32, Sales representative)

‘Normally she’ll do dry needling, soft tissue work, put the electrode pads on me, and then the adjustment. She’ll ice me down, or strap me, and by the end of that, that (migraine) pain has lessened. The neck adjustments are what I feel releases that migraine.’ (Participant 3, Age 27, Business development officer)

‘With dry needling, I found the most relief out of that, but also I saw the greatest relief from the adjustments.’ (Participant 4, Age 30, Quantity surveyor)

‘Releasing the (migraine) tension, and also the advice that they give you, like what to do when you feel stiff and how you should be sitting and your posture.’ (Participant 12, Age 29, Merchandising manager)

## Discussion

The study explored the physical impact and contributory factors of migraines on female adults and their experience of chiropractic treatment for migraine pain management. Theme 1 focused on the physical effects of migraines. The migraine headache usually occurs unilaterally, has a moderate to severe intensity, is throbbing or pulsating in character and can shift sides between or during attacks (Dodick 2018:1315; International Headache Society 2018:19). The present study revealed that migraines were severe, throbbing and pounding in character, and among two participants, it sometimes shifted sides during attacks. Additionally, neck pain was found among most participants. Migraines usually present with neck pain in the posterior cervical and trapezius regions (Dodick 2018:1315). Furthermore, 75% of migraineurs reported neck pain to be accompanied by their migraines (Bernstein et al. 2019:1).

A comprehensive study revealed that migraineurs experienced photophobia, phonophobia, nausea, giddiness or tinnitus (Vo et al. 2018:325). The available South African literature revealed that the physical symptoms experienced by the university student population in quantitative studies were photophobia, loss of appetite, nausea, dizziness, sleepiness, tingling, vomiting, tinnitus (Prangley 2010:iv), reduced concentration, lethargy and disrupted sleep patterns because of their migraines (Basdav et al. 2016:1680). Similarly, the present study is consistent with the aforementioned studies and previous literature (Connor et al. 2021:1; Peters et al. 2005:43; Vo et al. 2018:325). Migraine with aura can be accompanied by reversible visual symptoms such as zig-zag patterns, floaters, blind spots, flashes of light, scintillations or vision loss (International Headache Society 2018:20; Weatherall 2015:117). The participants in the present study had temporary visual symptoms, such as hazy or black spots, and zig-zag patterns. Other symptoms were photophobia, phonophobia, nausea, vomiting, dizziness and vertigo. The postdrome symptoms were lethargy and ‘feeling groggy’. This is consistent with the literature (Dodick 2018:1315; International Headache Society 2018:19).

The quality of life and individual functioning are substantially diminished among migraineurs (Dindo et al. 2015:109). Migraines often interfere with daily activities (Conner et al. 2019:1). The literature revealed that the impact on the ability to physically function during migraines included reduced concentration, impaired communication, difficulty in performing daily activities and resultant reduction in performing school or work activities (Basdav et al. 2016:1680; Estave et al. 2021:1009). Furthermore, studies revealed that migraines resulted in impaired sleep patterns, slow recovery and lost time (Nichols et al. 2017:4). Similarly, the participants in the present study also experienced these debilitating effects, including mental or physical incapacitation, lethargy and neglecting daily activities during their migraine attacks. Migraineurs perceived their limitations regarding daily activities, work, family and social life as a disability (Peters et al. 2005:44). Although migraines caused functional disability, most participants in the present study endured their symptoms to fulfil their work obligations.

Theme 2 focused on the factors contributing to migraines. There is a high comorbidity of migraines with stress, anxiety, depression and psychological disorders (Estave et al. 2021:1005; Dindo et al. 2015:109; Persson et al. 2021:3). The literature revealed that migraineurs described stress as a major contributory factor that triggered and intensified their migraines (Banciu & Bouleanu 2018:28; Connor et al. 2021:40; Pellegrino et al. 2018:1194). Similarly, in the present study, stress was a major contributing factor in triggering migraines.

Another contributing factor found among most participants was hormonal factors. Women who are susceptible to experiencing migraines are those that are sensitive to hormonal changes and may experience migraine with menstruation or pregnancy (Charles 2017:555; Pellegrino et al. 2018:1194; Ripa et al. 2015:780). Menstrual migraines are migraines that occur before or during menstruation in at least two out of three menstrual cycles (International Headache Society 2018:190). Menstrual migraines occurred among three quarters of the present study’s population. Furthermore, it is the most prevalent subtype of migraine among women (Todd, Lagman-Bartolome & Lay 2018:2). Menstruation was a triggering factor among 81% of migraineurs in 17 European countries (Vo et al. 2018:325). Additionally, menstrual migraines were found among 13% of Nigerian university students (Mustapha et al. 2019:43). In menstrual migraines or migraine without aura, migraineurs experienced a reduced migraine frequency during pregnancy because of hormonal fluctuations or high oestrogen levels. Similarly, the two participants in the present study who had menstrual migraine without aura, did not have migraines for the duration of their pregnancies and thus had an improvement in migraine frequency. In contrast, some women, especially with migraine with aura, experienced worsening of migraines (Alcantara & Cossette 2009:194). Supporting this finding, one participant with migraine with aura experienced severe migraines throughout pregnancy. Migraines reduce in frequency during menopause, because of the stabilising of hormones (Charles 2017:555; Ripa et al. 2015:774). It was reported that menopausal women with fluctuating hormones, hot flushes or night sweats had a higher migraine prevalence (Ripa et al. 2015:780). The two post-menopausal participants in the present study had a decrease in migraine frequency during and after menopause.

Oral contraceptive use has been shown to trigger and exacerbate migraines (Alcantara & Cossette 2009:194; Charles 2017:555; Ripa et al. 2015:780). Similarly, migraines were triggered by oral contraceptive use in the present study among all COC users (three participants). It is suggested that among those taking COC, the drop in oestrogen levels during the first few days of the menstrual cycle may contribute to triggering migraines (Allais et al. 2017:S85). In addition, migraines may also be triggered during the hormone-free interval in those on COC, thus causing more disabling migraines (Allais et al. 2017:S85; Machado et al. 2010:202; Todd et al. 2018:3). In the present study, two participants experienced migraines after the hormone-free interval (placebo week), and one participant had migraine attacks every week on the COC, including the hormone-free interval.

The dietary contributory factors found among the participants included cheese, chocolate, dairy, pungent food, caffeine, alcohol, dehydration and inconsistent eating habits. The above findings cohered with the available literature among migraineurs (Charles 2017:555; Connor et al. 2021:4; MacGregor 2017:ITC54; Moore et al. 2017:519; Vo et al. 2018:325). Weather-related factors and lighting, such as weather changes, humidity, sunlight, bright lights and fluorescent lights were found among the participants. These triggering factors are consistent in other studies (Moore et al. 2017:519; Pellegrino et al. 2018:1193; Vo et al. 2018:325).

There was insight into the awareness of the musculoskeletal factors that were contributing to the participants’ migraines. These were poor posture, forward head posture, poor ergonomics, physically demanding work, exercising, bruxism associated with temporomandibular joint dysfunction, muscular pain from travelling and overworking by sitting at a computer for long hours in a stationary position. Migraines are commonly aggravated by physical activity, such as walking or climbing stairs (International Headache Society 2018:19). Prior literature also revealed that muscle tension, shoulder tightness, physical activity, jaw dysfunction and neck stiffness were reported by migraineurs as musculoskeletal migraine triggering factors (Bernstein et al. 2019:1; Connor et al. 2021:7; Wayne et al. 2020:2).

Studies have documented the onset of migraines commonly during sleep or upon awakening (Dodick 2018:1315). Sleep deprivation or inconsistent sleeping habits were reported by a few participants in the present study as migraine triggers. It was revealed that migraineurs with poor sleep habits had a higher frequency of migraines, which may be attributed to poor sleep quality, duration and adopted coping behaviours such as sleeping too early to relieve migraines (Ferini-Strambi, Galbiati & Combi 2019:S107). To cope with their pain, most participants in the present study slept earlier than usual or during their migraine attacks. Previous studies reported that sleep deprivation, interrupted sleep, irregular or poor sleeping habits, and resultant mental and physical fatigue were also identified as migraine-triggering factors (Charles 2017:555; Moore et al. 2017:519; Mustapha et al. 2019:39; Pellegrino et al. 2018:1194; Vo et al. 2018:325).

Chiropractic treatment has been shown to reduce migraine frequency, duration, intensity and disability and has had favourable effects in reducing migraine medication use and frequency (Bernstein et al. 2019:4; Bryans et al. 2011:274; Clar et al. 2014:2; Rist 2019:532). Similarly, the present study cohered with these statements in the aforementioned studies. Regarding the effectiveness of chiropractic SMT on migraineurs’ quality of life, improvements were found relating to general health, physical condition and daily activity, with a 30.8% decrease in medication consumption in a South African clinical trial (Du Preez 2004:132). Another South African study also documented that chiropractic SMT decreased the disability, severity and the duration of migraines (Chopdat 2015:vii). Furthermore, chiropractic was considered by migraineurs as an effective approach to migraine management through the improvement of posture, flexibility and strength (Connor et al. 2021:5).

Participants expressed their appreciation for the chiropractor–patient relationship and considered it an important aspect to migraine management as it helped participants feel validated about their pain. Chiropractors in eThekwini reported to use the following treatment techniques for migraine patients: SMT, cervical traction, soft tissue therapy, stretching exercises, cryotherapy, electro-modalities, patient advice and education on stress management, exercise programmes and dietary advice (Kleingeld 2016:198). The findings of the present study cohered with all the above treatment techniques, and the participants additionally reported dry needling, the application of taping (strapping) and strengthening exercises as beneficial aspects of chiropractic care for migraines. It was documented that the majority of chiropractors in eThekwini expected the relief of their patients’ migraine symptoms within 2 days following treatment (Kleingeld 2016:198). Similarly, the participants in the present study found relief of their migraine symptoms and pain within the day of receiving chiropractic treatment or the following day. The participants sought chiropractic treatment for their migraines with the onset of their migraines, every 2 to 3 weeks, once a month or every few months. All the participants stated that chiropractic was beneficial in reducing their migraine pain and suffering.

## Limitations

The sample was restricted to only participants residing in the eThekwini region of KwaZulu-Natal in South Africa. Although the sample was small, information richness was still obtained.

## Recommendations

Future studies should include more qualitative research studies on the effects of migraines on the physical functioning and quality of life among the female population. The impact of menstruation, pregnancy and menopause on migraines should be further investigated in other studies. A more detailed study should be undertaken to ascertain the impact and efficacy of chiropractic treatment for migraines.

## Conclusion

The majority of the participants experienced moderate to severe chronic migraines and migraines without aura. During their migraine attacks, the participants experienced debilitating effects, felt mentally or physically incapacitated, described that their life came to a standstill, were unable to complete daily activities and had a resultant decreased quality of life. The study highlighted that chiropractic treatment was favourable among the female population in improving the quality of life and reducing the severity, disability, duration of suffering and frequency of migraines. The study builds on the knowledge of the detrimental effects of migraines on physical functioning, with participant experiences confirming the use of chiropractic as an effective treatment approach for migraine pain management. The findings of the study contribute to a greater awareness of chiropractic as an effective evidence-based treatment approach for migraine pain management, which may be beneficial to migraineurs and healthcare practitioners seeking to co-manage patients.
